# CAR-T cell therapy in triple-negative breast cancer: Hunting the invisible devil

**DOI:** 10.3389/fimmu.2022.1018786

**Published:** 2022-11-22

**Authors:** Fatemeh Nasiri, Mehrasa Kazemi, Seyed Mohamad Javad Mirarefin, Maral Mahboubi Kancha, Milad Ahmadi Najafabadi, Faeze Salem, Setareh Dashti Shokoohi, Sahar Evazi Bakhshi, Pouya Safarzadeh Kozani, Pooria Safarzadeh Kozani

**Affiliations:** ^1^ Department of Medical Biotechnology, Faculty of Medical Sciences, Tarbiat Modares University, Tehran, Iran; ^2^ Department of Production Platforms & Analytics, Human Health Therapeutics Research Centre, National Research Council Canada, Montreal, QC, Canada; ^3^ Department of Laboratory Medicine, Thalassemia Research Center, Hemoglobinopathy Institute, Mazandaran University of Medical Sciences, Sari, Iran; ^4^ Department of Immunology, Faculty of Medical Sciences, Tarbiat Modares University, Tehran, Iran; ^5^ Department of Medical Nanotechnology, School of Medicine, Shahroud University of Medical Sciences, Shahroud, Iran; ^6^ Department of Anatomical Sciences, School of Medicine, Guilan University of Medical Sciences, Rasht, Iran; ^7^ Department of Medical Biotechnology, Faculty of Paramedicine, Guilan University of Medical Sciences, Rasht, Iran

**Keywords:** triple-negative breast cancer, chimeric antigen receptor, solid tumors, cancer immunotherapy, adoptive cell therapy, T-cell therapy

## Abstract

Triple-negative breast cancer (TNBC) is known as the most intricate and hard-to-treat subtype of breast cancer. TNBC cells do not express the well-known estrogen receptor, progesterone receptor, and human epidermal growth factor receptor 2 (HER2) expressed by other breast cancer subtypes. This phenomenon leaves no room for novel treatment approaches including endocrine and HER2-specific antibody therapies. To date, surgery, radiotherapy, and systemic chemotherapy remain the principal therapy options for TNBC treatment. However, in numerous cases, these approaches either result in minimal clinical benefit or are nonfunctional, resulting in disease recurrence and poor prognosis. Nowadays, chimeric antigen receptor T cell (CAR-T) therapy is becoming more established as an option for the treatment of various types of hematologic malignancies. CAR-Ts are genetically engineered T lymphocytes that employ the body’s immune system mechanisms to selectively recognize cancer cells expressing tumor-associated antigens (TAAs) of interest and efficiently eliminate them. However, despite the clinical triumph of CAR-T therapy in hematologic neoplasms, CAR-T therapy of solid tumors, including TNBC, has been much more challenging. In this review, we will discuss the success of CAR-T therapy in hematological neoplasms and its caveats in solid tumors, and then we summarize the potential CAR-T targetable TAAs in TNBC studied in different investigational stages.

## Introduction

Breast cancer is the malignancy arising from the breast tissues. It is the most common malignancy worldwide and is estimated to be detected in more than 280,000 women in the United States in 2022 (1). According to the estimations, more than 43,000 women will die because of breast cancer only in 2022 (1). The molecular process that leads to the emergence of breast cancer entails the active proliferation of epithelial cells of the breast tissue resulting in the formation of malignant cells in the ductal or lobular compartment of the breast (2, 3). There are various types of classifications for categorizing breast cancer. Histologic grade, disease stage, and expression of classic hormone and growth factor receptors are three well-known criteria used for breast cancer classification and determining the aggressive capacity of a breast tumor (2–7). The hormone and growth factor receptors employed for breast cancer classification include estrogen receptor (ER), progesterone receptor (PR), and human epidermal growth factor receptor 2 (HER2) (2–7). Based on the expression or lack of expression of these receptors and the expression rate of Ki-67, there are four subtypes of breast cancer including Luminal A, Luminal B, HER2 amplified, and triple-negative breast cancer (TNBC) (2–7). The first three subtypes are easier to manage since the patients with these subtypes are eligible for antibody-based therapies such as anti-HER2 monoclonal antibodies (mAbs) or antibody-drug conjugates (ADCs), tyrosine kinase inhibitors, and endocrine therapies (including ER degraders) (8–10). Dissimilar from these subtypes, TNBC is a heterogeneous type of basal-like tumor that does not express the abovementioned receptors and it represents about 15 to 20% of all breast cancer cases (11).

Due to the unresponsiveness of TNBC patients to anti-HER2 mAb-based and endocrine therapies, traditional anticancer treatments including surgery, radiotherapy, and chemotherapy continue to be the commonly available therapies for these patients (12, 13). Of note, these traditional cancer treatment modalities manage to eradicate malignant cells in a nonspecific manner resulting in mild to severe treatment-related side effects (13). Additionally, poly (ADP-ribose) polymerase (PARP) inhibitors are a class of pharmacological inhibitors that have been approved by the US Food and Drug Administration (FDA) for the treatment of TNBCs with BRCA mutations (14). Recently, many bodies of research have indicated that the immune system pathways are remarkably involved in the emergence and progression of TNBC (15, 16). In this case, researchers have utilized immune checkpoint blockade therapies such as programmed death-1 (PD-1)- or programmed death-ligand 1 (PD-L1)-specific antibodies for suppressing the outgrowth of TNBC (16, 17). Moreover, T-cell-redirecting bispecific antibodies (TRBAs) have also been under investigation as candidates for the treatment of TNBC (16, 17). Recently, the US FDA approved the PD-L1-specific mAb *atezolizumab* in combination with the chemotherapeutic agent *nab-paclitaxel* for the treatment of patients with PD-L1-proficient unresectable TNBC (18). Such progress in the field of TNBC immunotherapy has encouraged researchers to employ other types of cancer immunotherapy such as cancer vaccines and adoptive cell therapy using genetically engineered immune cells for a more selective and successful fight against TNBC (16, 19, 20).

**Figure 1:**
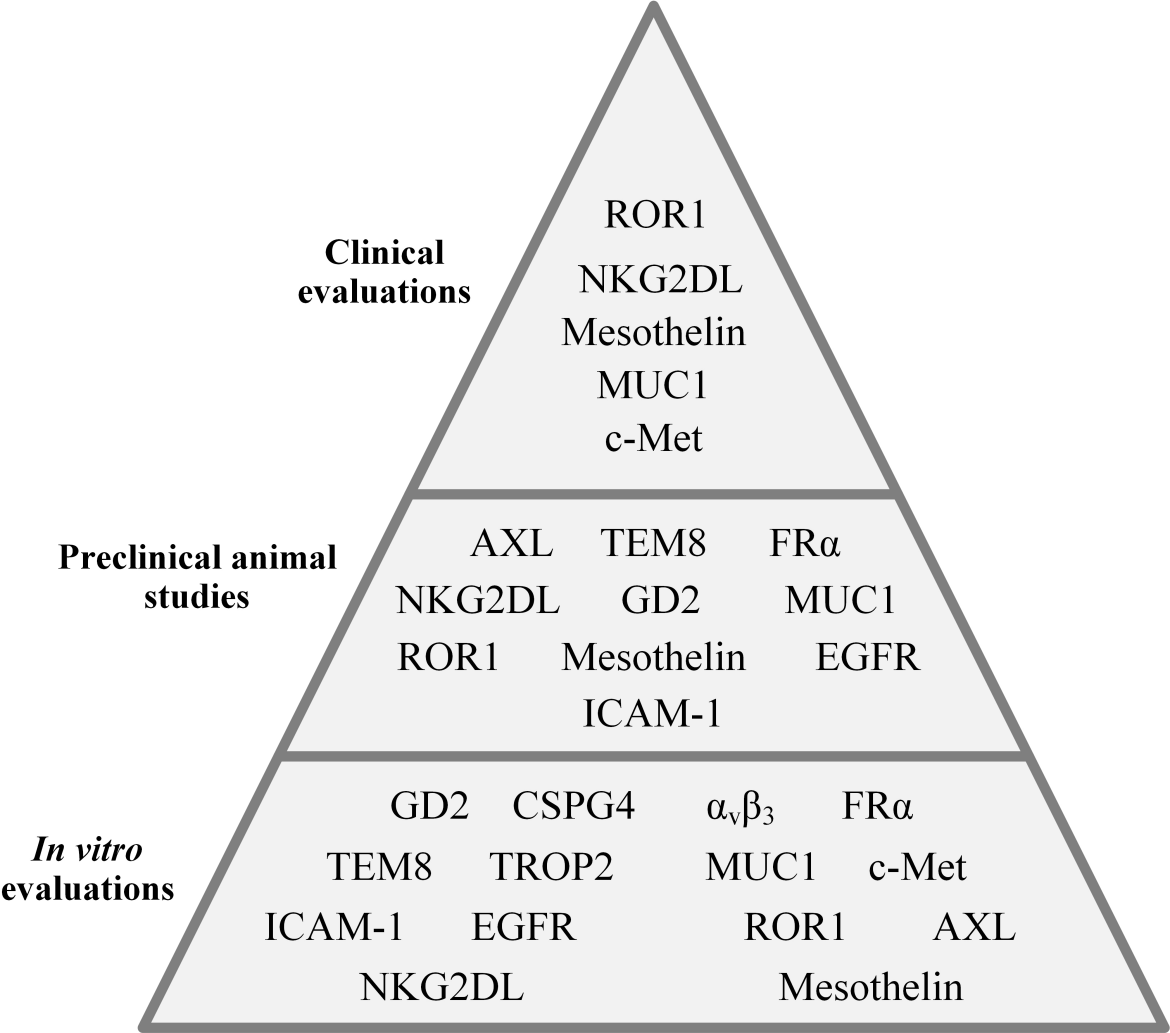
An overall presentation of the CAR-T-targeted TNBC-associated antigens evaluated in different investigational stages.

The importance of cancer-specific immune cells in TNBC was elucidated when researchers discovered that the presence of tumor-infiltrating lymphocytes (TILs) in residual disease following neoadjuvant chemotherapy is correlated with superior prognosis in TNBC patients (21). In a population of TILs, there are tumor-reactive T cells that recognize cancer-specific antigens and are responsible for the antitumor activity of a TIL population (22, 23). However, there are several limitations in regards to the application of TILs for adoptive cell therapy of various types of tumors. These caveats include the exhausted phenotype of TILs resulting from multiple target antigen encountering, and difficulties in regards to the isolation and *ex vivo* expansion of TILs (24, 25). Genetically engineered T cells expressing chimeric antigen receptors (CAR-Ts) have recently changed the face of immune cell-based cancer immunotherapy (26, 27). CAR-Ts possess antibody-derived targeting domains linked to T-cell activating domains which grant them the ability to recognize cancer cells *via* target cell membrane-expressed antigens independent of major histocompatibility complexes (MHCs) (26, 27). Moreover, CAR-Ts can be easily expanded to clinically relevant scales; therefore, they are considered an ideal alternative to TILs (26, 27). The major purpose of this review is to discuss CAR-T therapy hurdles in solid tumors with a specific focus on TNBC, shine a light on the crucial stratagems to tackle these caveats, and discuss the potential and promising target antigens under investigation in various stages, from early developmental stages to clinical settings, for CAR-T therapy of TNBC (**Figure 1**).

## CAR-T therapy fundamentals

About twenty years ago, when a person was diagnosed with cancer, surgery, chemotherapy, and radiotherapy were the only available treatment options. However, these treatment modalities could not mediate complete disease remission in most cases (28–30). Additionally, disease relapse, shortly after the completion of the treatment, was always a challenge (28–30). Alongside the mentioned limitations, the severity of the treatment-related side effects was another factor affecting the patients’ general well-being (28–30). Years later, with the emergence of immunotherapy and the development of novel treatment modalities, cancer treatment became more selective and efficient resulting in prolonged survival and reduced adverse events in patients.

CAR-Ts are the ultimate result of years of research and experience in various aspects of immunotherapy and cell therapy. The process of CAR-T generation starts with obtaining peripheral blood mononuclear cells (PBMCs) from patients (in the case of autologous CAR-T therapy) or third-party healthy donors (in the case of allogeneic CAR-T therapy) (31, 32). From the population of the isolated PBMCs, T lymphocytes are isolated. These T cells undergo activation and genetic manipulation steps, usually using retroviruses, to express a synthetic chimeric antigen receptor (CAR) on their surface (32). After expansion to a number of cells required for clinical applications, the engineered T cells are infused into recipients. It is worth mentioning that before CAR-T administration, patients usually undergo lymphodepletion chemotherapy (32).

A CAR construct is made of three important sections. An extracellular domain, a transmembrane domain, and an endodomain (32). The ectodomain of CARs is derived from a tumor antigen-specific fragment of a mAb such as a single-chain fragment variable (scFv) or single variable domain on a heavy chain (V_HH_) (32, 33). This targeting domain is linked to a flexible spacer, called a *hinge*, acting as a connector between the ectodomain and the transmembrane domain (32). Recently, it has been demonstrated that both hinge and transmembrane domains have remarkable impacts on CAR expression and signaling (34–36). In detail, the CAR expression rate on T cells and its stability are highly impacted by the type of the transmembrane domain (rather than hinge) (34). The CAR transmembrane domain can control the extent of CAR signaling by regulating the level of CAR surface expression (34). On the other hand, the hinge domain can control the membrane transport efficiency of CARs in T cells as well as the modality of CAR expression (34). Muller et al. demonstrated that CAR constructs with CD28 (but not CD8) as the transmembrane domain can form heterodimers with the endogenous CD28 present in human T cells which can result in more robust signal transductions enhancing CAR-T activation in the context of confronting low levels of a particular target antigen (35). They added that the CD28 transmembrane domain is capable of regulating various CAR-T functionalities by employing endogenous pathways (35). Additionally, June et al. reported that CAR-Ts with CD28 transmembrane domain exhibited prolonged *in vitro* expansion (up to 3 months) after a single TCR stimulation and without any IL-2 supplementation (37). Other researchers have added that CAR-Ts whose hinge and transmembrane domain are both based on CD28 are capable of producing target antigen-dependent inflammatory cytokines more than CAR-Ts whose hinge and transmembrane domain are both based on CD8 (38). Moreover, it has been demonstrated that these CAR-Ts require a lower antigen density for activation (studied only in CD19-redirected CAR-Ts with 4-1BB as the co-stimulatory domain) (39). Conclusively, broader investigation into the impact of CAR hinge and transmembrane domains on the persistence, tumoricidal efficacy, and phenotypic characteristics of CAR-Ts can further help the development of CAR-Ts with greater therapeutic benefits.

The endodomain of CARs is responsible for activating the effector cell upon target antigen encountering. This part of CARs has a CD3ζ signaling domain derived from the CD3 complex of the T-cell receptor (TCR) fused to intracellular costimulatory domains such as CD27, CD28, 4–1BB, OX40, and/or ICOS (32). Of note, dissimilar from first-generation CAR-Ts which did not possess any intracellular costimulatory domains, second- and third-generation CAR-Ts have one and two intracellular costimulatory domains, respectively (40–43). According to scientific evidence, the addition of intracellular costimulatory domains to the construct of CARs resulted in superior post-infusion expansion and persistence of second- and third-generation CAR-Ts in comparison with their first-generation counterparts (40–43). Recently, the construct of CARs has been further modified to achieve certain aims during CAR-T therapy. For instance, fourth-generation CAR-Ts possess an intracellular expression inducer of a cytokine of interest resulting in CAR-T-mediated tumor site delivery of a cytokine of interest leading to more enhanced and safer antitumor responses, especially in solid tumor CAR-T therapy (44–46). In detail, one of the well-known characteristics of solid tumors is the heterogeneity of tumor cells. Various populations of tumor cells might express different antigens in which case a single type of CAR-Ts redirected against a particular type of target antigen may not be clinically beneficial. However, expanding the antitumor responses by triggering the immune responses by other types of endogenous immune cells can help mediate more efficient tumoricidal reactions. In this regard, CAR-Ts have been engineered to specifically deliver a transgenic product, which can be chemokines or cytokines, to the targeted tumor sites (44, 46, 47). In detail, Chmielewski et al. generated CAR-Ts harboring an engineered intracellular expression inducer of IL-12 which can recruit macrophages (47). These CAR-Ts, which are also regarded as “*T-cell redirected for universal cytokine-mediated killing (TRUCKs)*” or “*armored CAR-Ts*”, are engineered to produce and release inducible IL-12 upon encountering CAR target antigen (47). This mechanism results in simultaneous antitumor attacks against both tumor cells expressing the CAR-redirected target antigen and tumor cells deficient in its expression (47). This strategy demonstrated that local delivery of IL-12 by CAR-Ts in the solid tumor tissues can be applied for targeting both target antigen-negative and hard-to-reach tumor cells through recruiting and activating innate immune components without the risk of systemic cytokine administration-related toxicities (47). Such CAR-Ts can be clinically valuable for targeting different target antigens of TNBC.

**Figure 2:**
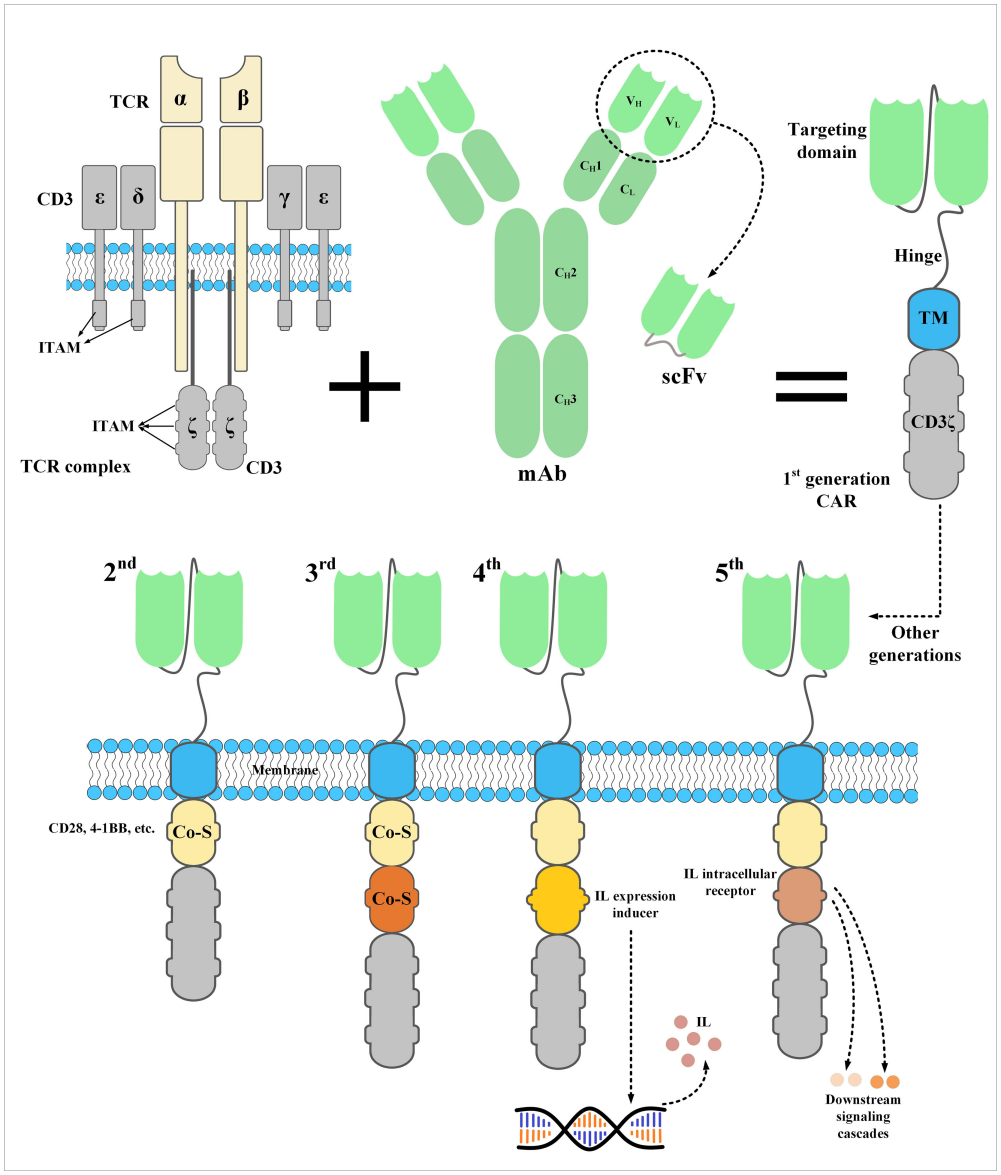
The structure of a CAR and its five generations. CARs are the result of meticulous protein engineering. The targeting domain of CARs is usually derived from the single-chain fragment variable (scFv) of a monoclonal antibody. scFvs are made from the variable light chain (V_L_) and variable heavy chain (V_H_) of a monoclonal antibody fused together through a synthetic linker peptide. A spacer called *hinge* connects the targeting domain of CARs to their transmembrane domain, which connects the ectodomain to the endodomain. Currently, the endodomain of CARs consists of one or two costimulatory domains and an activation domain. An interleukin expression inducer domain and an interleukin intracellular receptor are also located on the endodomain of the fourth- and fifth-generation CARs, respectively. Of note, the first-generation CARs lacked a costimulatory domain which led to their inadequate *in vivo* persistence and weak antitumor responses. CAR, chimeric antigen receptor; Co-S, costimulatory domain; IL, interleukin; ITAM, immunoreceptor tyrosine-based activation motif; mAb, monoclonal antibody; scFv, single-chain fragment variable; TCR, T-cell receptor; TM, transmembrane domain.

Moreover, fifth-generation CAR-Ts have also been developed which harbor an intracellular domain of a cytokine receptor, for example, interleukin (IL) 2 receptor subunit beta (IL-2Rβ) (44–46). In detail, proper activation and expansion of T cells are highly dependent on various signals including T-cell receptor (TCR), co-stimulatory, and cytokine-mediated signals (48). In the case of CAR-Ts, T cells can only benefit from TCR signaling which is provided through their CD3ζ domain and co-stimulatory domains (48). Therefore, researchers have generated CAR-Ts with novel constructs which are capable of inducing cytokine signaling once the effector cells encounter the target antigen (48). Fifth-generation CAR-Ts harbor truncated cytoplasmic domain of the IL-2Rβ and a STAT3-binding tyrosine-X-X-glutamine (YXXQ) motif alongside a primary activation domain and a co-stimulatory domain (48). Kagoya et al. reported that these CAR-Ts demonstrated target antigen-specific activation of the JAK kinase and the STAT3 and STAT5 transcription factor signaling pathways (48). These signaling pathways prevented terminal phenotypic differentiation of the effector cells and mediated their efficient expansion *in vitro* (48). Moreover, *in vivo* assessments proved that these CAR-Ts function efficiently in terms of persistence and tumoricidal reactions in comparison with their conventional counterparts, both in hematologic and solid tumors (48). Such novel CAR constructs can be evaluated in the case of TNBC CAR-T therapy to help achieve more efficient and less toxic antitumor responses. The constructs of different generations of CARs have been illustrated in **Figure 2**.

In regards to the application of scFvs as the targeting domain of CARs, it has been discovered that target antigen-independent CAR scFv aggregation occurs which is a result of CAR CD3ζ phosphorylation and/or the intrinsic instability of the V_H_ and V_L_ chains of the scFv (49). This phenomenon, known as “*tonic signaling*”, can mediate premature CAR-T activation (before encountering the intended target antigen) and render these effector cells exhausted leading to their impaired antitumor activity (49). Long et al. have demonstrated that the 4-1BB co-stimulatory domain in CAR-Ts better manages to minimize the phenotypic characteristics of CAR-T exhaustion mediated by the aggregation of CAR scFvs in comparison with CAR-Ts harboring CD28 as their co-stimulatory domains (49). These findings are consistent with the reports by Frigault and colleagues who reported that antigen-independent signaling cascades in CAR-T with CD28 as their co-stimulatory domains culminate in their exhaustion (based on animal studies) (37). Such findings might explain why CAR-Ts with the 4-1BB co-stimulatory domain demonstrate superior post-infusion persistence over their counterparts with the CD28 co-stimulatory domain (49).

Alongside the mentioned findings, other researchers have proposed the replacement of particular framework residues of the CAR scFv as an attempt to minimize the aggregation tendency of these scFvs and circumvent spontaneous tonic signaling (50). In detail, Landoni et al. employed *in silico* techniques to find the framework residues of a CSPG4-specific scFv (known as 763.74 scFv) that contributed to its stability and whose substations would result in decreasing the tendency of scFv aggregation, and consequently tonic signaling and CAR-T exhaustion (50). Moreover, the importance of scFv is also accentuated in regard to the on-target off-tumor effects of CAR-Ts. In the context of solid tumors, target antigens are usually expressed by healthy tissues as well (however, at physiological levels), which results in the CAR-T-mediated cytolytic reactions against healthy cells (51). Since the discovery of tumor-specific antigens is not always a feasible task, researchers have suggested targeting the tumor-specific glycoforms (called T, Tn, or sialyl Tn glycoforms) of commonly known TAAs (such as MUC1) (51). mAbs against such glycoforms are called cancer-specific mAbs (CasMabs) and scFvs derived from these CasMabs could be applied as the targeting domain of CAR-Ts for the development of cancer-specific CAR-Ts (Cas-CAR-Ts) (51). Such CAR-Ts have been developed and assessed in different investigational stages, and have proven to be efficient and safe (51). As another scFv-related strategy to minimize off-tumor toxicities, researchers have demonstrated that CAR-Ts equipped with targeting domains whose affinity towards their target antigen is moderate (micromolar affinity range), rather than high (nanomolar affinity range), manage to efficiently target tumor cells with high target antigen density while sparing normal cells which express the target antigen at a lower level (52). All of the mentioned strategies accentuate the importance of CAR targeting domains, particularly scFvs, and how their engineering can culminate in the development of safer and more efficient CAR-Ts (52).

## CAR-T therapy challenges in solid tumors

The tremendous success of CAR-T therapy in relapsed/refractory (R/R) B-cell hematologic malignancies has rendered this type of cancer treatment approach a considerable treatment option (53–55). Based on such favorable clinical outcomes especially in patients non-responsive to the other types of treatments, the US FDA has approved six CAR-T products for the treatment of patients with failed previous lines of treatments. The list of the FDA-approved CAR-T products has been summarized in **Table 1**. However, patients with several hematologic malignancies, including T-cell neoplasms, as well as solid tumor patients have not yet benefited from the anticancer capability of this unique type of therapy (68–70). There are various limitations attributed to the inability of CAR-Ts to mediate tumor rejection and disease remission in solid tumors. In detail, CAR-Ts are redirected towards TAAs or TSAs (51, 71). Therefore, ideal CAR-T targets are antigens that are absent or have a low-level expression on the healthy cells of normal tissues but are overexpressed by malignant cells (51, 71). Targeting such target antigens can result in minimal unwanted off-tumor toxicities toward healthy tissues. However, finding target antigens with such characteristics is hardly feasible in solid tumors (51, 71). Additionally, another limitation of solid tumor CAR-T therapy is intratumor antigen heterogeneity described as tumor cells of a particular type of tumor expressing different levels of a CAR-redirected target antigen on their surface or not expressing the target antigen at all (72–74). This phenomenon results in impaired detection of malignant cells by CAR-Ts further leading to disease escape and relapse (72–74).

**Table 1:** A list of CAR-T products approved by the US FDA for the treatment of different hematologic malignancies.

Generic name	Brand name	Year of first Approval	Target (targeting domain type)	Generation (intracellular signaling domain)	Indication(s) (line of treatment approved for)	Reference
Tisagenlecleucel	Kymriah^®^	2017	CD19 (scFv)	2^nd^ (4-1BB-CD3ζ)	B-ALL (3^rd^ line) DLBCL (3^rd^ line) FL (3^rd^ line)	(56–67)
Axicabtagene ciloleucel	Yescarta^®^	2017	CD19 (scFv)	2^nd^ (CD28-CD3ζ)	DLBCL (2^nd^ line) FL (3^rd^ line)	
Brexucabtagene autoleucel	Tecartus^®^	2020	CD19 (scFv)	2^nd^ (CD28-CD3ζ)	MCL (3^rd^ line) B-ALL (3^rd^ line)	
Lisocabtagene maraleucel	Breyanzi^®^	2021	CD19 (scFv)	2^nd^ (4-1BB-CD3ζ)	DLBCL (3^rd^ line)	
Idecabtagene vicleucel	Abecma^®^	2021	BCMA (scFv)	2^nd^ (4-1BB-CD3ζ)	MM (5^th^ line)	
Ciltacabtagene autoleucel	Carvykti^®^	2022	BCMA (single-domain antibody)	2^nd^ (4-1BB-CD3ζ)	MM (5^th^ line)	

B-ALL, B-cell acute lymphoblastic leukemia; DLBCL, diffuse large B-cell lymphoma; FL, follicular lymphoma; MCL, mantle cell lymphoma; MM, multiple myeloma; scFv, single-chain fragment variable.

Another limitation of CAR-T therapy in solid tumors is associated with the immunosuppressive tumor microenvironment (TME). The TME consists of various types of immune suppressor cells such as regulatory T cells (Tregs), myeloid-derived suppressor cells (MDSCs), cancer-associated fibroblasts (CAFs), and tumor-associated macrophages (TAMs) (75–78). There are also various suppressive factors such as chemokines, cytokines, and extracellular matrix (ECM) present in the TME. The immune suppressor cells residing in the TME are beneficial for tumors in terms of supporting their progression, angiogenesis, and metastasis by providing them with various types of growth factors, chemokines, and cytokines such as ILs, transforming growth factor beta (TGF-β), indoleamine 2,3-dioxygenase (IDO), and vascular endothelial growth factor (VEGF) (75–78). Moreover, the expression of immune checkpoint molecules (such as CTLA-4 and PD-1) on tumor-residing T lymphocytes is another factor that hampers antitumor reactions (79–82). Overall, the mentioned immunosuppressive factors in the TME remarkably suppress CAR-T-mediated antitumor reactions in solid tumor CAR-T therapy.

**Figure 3:**
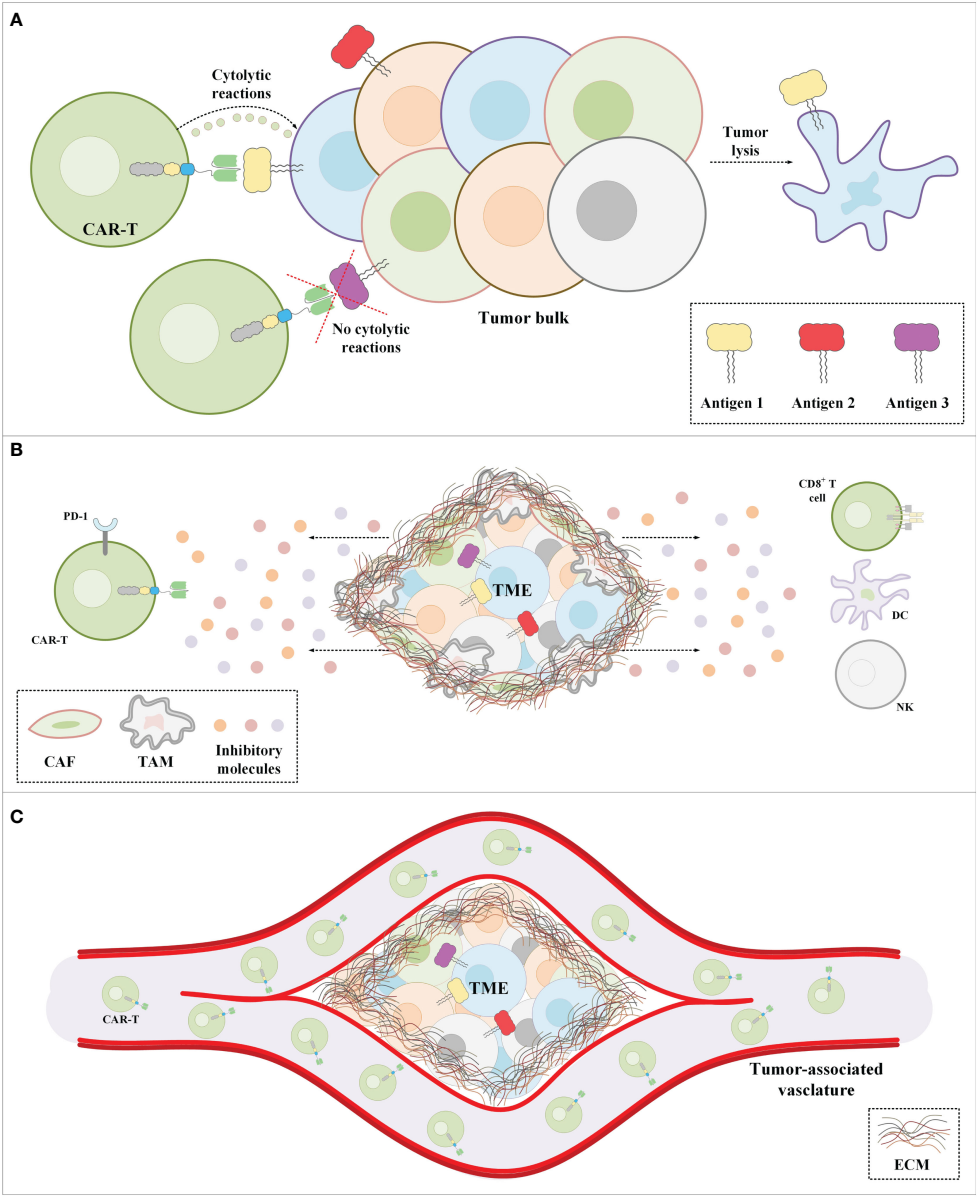
The major impediments of CAR-T therapy in solid tumors. **(A)** CAR-Ts encounter tumor cells only a proportion of which express the CAR-redirected target antigen. Moreover, in a population of tumor cells, there might be malignant cells not expressing any known target antigens. **(B)** The immunosuppressive nature of the TME suppresses CAR-T antitumor activity. **(C)** The extracellular matrix of solid tumors is the most important physical barrier between the tumor cells and CAR-Ts in CAR-T therapy of solid tumors. Cancer-associated fibroblasts are among the mediators responsible for the formation of the stroma extracellular matrix. CAF, cancer-associated fibroblast; CAR-T, chimeric antigen receptor T-cell; DC, dendritic cell; EMC, extracellular matrix; NK, natural killer cell; PD-1, programmed death-1; TAM, tumor-associated macrophage; TME, tumor microenvironment.

In addition to all of the abovementioned hurdles, the inadequate trafficking and infiltration of CAR-Ts into the tumor tissues is known as another major limitation impairing the functionality and tumoricidal activity of CAR-Ts (83–86). Dissimilar to CAR-T therapy in hematologic malignancies where CAR-Ts encounter target cells in the bloodstream and the lymph nodes, in the case of solid tumors, CAR-Ts must cross the vascular endothelium and penetrate the tumor tissues (86). There are various tumor-associated action mechanisms that remarkably limit the accessibility of CAR-Ts to tumor cells (83–86). In brief, CAR-T tumor site penetration and trafficking are substantially correlated with the presence of various types of chemokines (87). Tumor tissues downregulate the expression of such chemokines leading to restricted tumor tissue CAR-T infiltration (87). Additionally, researchers demonstrated that the dense nature of the ECM of the tumor tissues is an important obstacle to CAR-T penetration and infiltration into the TME (88). Tumor ECM also protects the TME from the antitumor activity of CAR-Ts as it has been demonstrated that its degradation can result in superior CAR-T-mediated tumor cell eradication (89). In a nutshell, profound knowledge of solid tumor mechanisms can better help us develop counterstrategies to overcome the mentioned hurdles for a more efficient and safer CAR-T therapy in patients with various types of solid tumors. A simplified view of the principal limitations of CAR-T therapy in solid tumors and examples of counterstrategies for tackling these hurdles have been brought together in **Figure 3** and **Table 2**, respectively.

**Table 2:** Significant limitations related to CAR-T therapy of solid tumors and examples of strategies to tackle these challenges.

Limitations	Examples of strategies
CAR-T target antigen heterogeneity or lack of target antigen specificity	* TRBA-secreting CAR-Ts (90) * TRBA therapy (91) * TRBA-expressing oncolytic viruses (92, 93) * Oncolytic vaccinia virus-mediated induction of CAR-T target antigen expression (94–96) * Bispecific CAR-Ts (97) * Trivalent CAR-Ts (98, 99) * CAR-T cocktail therapy (100) * Screening for alternative target antigens (71)
Immunosuppressive TME	* Tackling the hypoxic TME nature (101–104) * Metabolic reprogramming of CAR-Ts (105)
*In vivo* CAR-T exhaustion	* Immune checkpoint blockade therapy (106) * CAR cells with PD-1 disruption (107) * CAR-T cells that secrete blocking antibodies against CTLA-4 and PD-1 (108, 109)
Poor tumor site CAR-T trafficking	* Regional delivery of CAR-Ts (110–112) * CAR-Ts that express chemokine/cytokine receptors (113–115) * Blocking protein kinase A localization (116) * Photothermal therapy (117, 118) * Using antiangiogenic drugs (119–122)
Inadequate post-infusion persistence	* *Ex vivo* phase media optimization (123–130) * CAR-T activity boosting vaccines (131–136)

CAR-T, chimeric antigen receptor T-cell; TME, tumor microenvironment; TRBA, T-cell-redirecting bispecific antibody.

## Targets antigens investigated for the CAR-T therapy of TNBC

### Chondroitin sulfate proteoglycan 4 (CSPG4)

CSPG4 is a cell membrane-spanning protein with a high level of glycosylation (137). Various roles have been proposed for CCSPG4 which include involvement in the regulation of neuronal networks, replacement of epithelial keratinocytes, and homeostasis of epidermal stem cells (137). CSPG4 has been studied as a target antigen in cancer immunotherapy based on its limited expression level in normal tissues and overexpression and supporting roles in cancer progression and invasion in various types of neoplasms, including TNBC (137). Researchers demonstrated that antibody-mediated targeting of CSPG4 using an scFv fused to Tau, which is a negative regulator of protein translation, results in efficient cytotoxicity against CSPG4-proficient TNBC-derived cell lines including MDA-MB-231 and Hs 578T (138).

In 2014, Geldres et al. generated second-generation CSPG4-redirected CAR-Ts, and reported that these cells significantly suppressed the growth of various CSPG4-expressing cell lines (including SENMA, CLB, UACC-812, MILL, MDA-MB-231, PHI, and PCI-30) (139). Moreover, these researchers reported that these CAR-Ts suppressed tumor cell growth in human melanoma, head and neck squamous cell carcinoma, and breast carcinoma preclinical mouse models established using SENMA, PCI-30, and UACC-812 cell lines, respectively (139). In the same year, Beard et al. generated second-generation CSPG4-redirected CAR-Ts using murine-based scFvs and reported target antigen-dependent cytotoxicity and cytokine secretion of these CAR-Ts against glioblastoma, breast cancer, mesothelioma, osteosarcoma, and melanoma CSPG4-expressing cell lines (A1207, MDAMB231, Mill, MgG-63, and mel938, respectively) and glioblastoma stem cells (140). Such data may support the applicability of CSPG4-redirected CAR-Ts in TNBC and various other neoplasms. However, more preclinical and clinical data are critically required for more reliable conclusions in this regard.

### Intracellular adhesion molecule-1 (ICAM-1)

ICAM-1 is a transmembrane glycoprotein that mediates the transmigration of leukocytes through the endothelium of various cell types (141). In 2014, Guo et al. reported that ICAM-1 is upregulated and overexpressed in TNBC cell lines and tissues and it might act as a possible biomarker and target antigen for the diagnosis and treatment of TNBC (142). These researchers demonstrated that antibody-assisted targeting of ICAM-1 is feasible and efficient even after systemic administration into xenograft TNBC tumor models (142). In 2017, Park et al. generated ICAM-1-redirected CAR-Ts with micromolar affinity against ICAM-1 (instead of nanomolar affinity) to prevent CAR-T-mediated cytotoxicity in non-malignant cells with normal ICAM-1 expression levels (52). These researchers demonstrated that these affinity-tuned CAR-Ts demonstrated superior tumoricidal efficacy and safety index compared to their higher affinity counterparts (52). Moreover, in 2019, using preclinical models, it was demonstrated that micromolar affinity-tuned ICAM-1-redirected CAR-Ts can target tumors with a high level of ICAM-1 expression while sparing normal tissues with lower and basal ICAM-1 expression levels with significant efficacy (143). The researchers of this report also suggested the investigation of micromolar affinity-tuned ICAM-1-redirected CAR-Ts in a Phase I clinical trial for assessing the safety and possible efficacy of these cells against R/R thyroid cancers (143). Moreover, Yang et al. generated ICAM-1-redirected CAR-Ts and investigated the efficacy of these cells *in vitro* and *in vivo* (144). These researchers demonstrated that *in vitro* CAR-T-mediated cytotoxicity against HeLa and MDA-MB-231 cell lines was specific and ICAM-1 expression-dependent (144). However, the ongoing preclinical assessments of this study, from which no results have yet been reported, will further elucidate the applicability and safety index of targeting ICAM-1 using CAT-Ts for the treatment of TNBC (144).

### Natural killer group 2, member D ligand (NKG2DL)

Malignant cells, such as TNBC cells, exhibit upregulated levels of stress-induced ligands, which are naturally recognized by molecules such as natural killer group 2, member D (NKG2D) (145). NKG2DL has been considered an immunotherapy target antigen in various studies (146). In 2005, Zhang et al. generated CARs by fusing the full-length NKG2D to the cytoplasmic domain of CD3ζ and endogenous DAP10 costimulation (146). These researchers demonstrated that T cells expressing these CARs reacted to NKG2DL-expressing tumor cells by secreting cytokines and chemokines and exhibiting cytotoxicity (146). Moreover, *in vivo* results also supported the tumor suppression ability of these CAR-Ts (146). Other researchers have also reported that NKG2DL-redirected CAR-Ts mediated disease remission in a patient with acute myeloid leukemia (AML) (NCT02203825) (147, 148).

In 2018, Han et al. evaluated the antitumor activity of NKG2DL-redirected CAR-Ts against TNBC cell lines and cell line-established preclinical TNBC mouse models (149). These researchers generated different NKG2DL-redirected CAR-Ts by fusing the extracellular domain of human NKG2D to the TCR CD3ζ alone or with CD27 or 4-1BB co-stimulatory domains (149). They reported that the *in vitro* expansion of CAR-Ts without any co-stimulatory domain was dependent on the high CD25 expression and the presence of IL-2 (149). Moreover, it was reported that NKG2DL-redirected CAR-Ts efficiently recognized TNBC NKG2DL-expressing MDA-MB-231 and MDA-MB-468 cell lines and eliminated them (149). *In vivo* experiments demonstrated tumor growth suppression ability of NKG2DL-redirected CAR-Ts in MDA-MB-231-derived TNBC preclinical mouse models paving the way for more preclinical and early phase clinical evaluations of this potential TNBC treatment approach (149). It is worth mentioning that a Phase I clinical trial (NCT04107142) has investigated the safety and tolerability of NKG2DL-redirected CAR-Ts in patients with R/R solid tumors including TNBC; however, the results are yet to be reported.

### Receptor tyrosine kinase AXL

AXL is a well-known member of the *TAM* family of receptor tyrosine kinases. Growth arrest-specific protein 6 (GAS6) is known as a high-affinity ligand for AXL (150, 151). AXL signaling operates as a critical pathway mediating tumor cell survival, expansion, migration, and invasion suggesting the potential of AXL as a suitable cancer treatment target antigen (150, 151). Normally, AXL has a low expression level during adulthood; however, its abnormal expression has been observed in various types of neoplasms including breast cancer (152).

Ye et al. developed an anti-AXL mAb with the ability to target both human and murine AXL (153). These researchers demonstrated that this mAb suppressed tumor outgrowth, reduced distant organ tumor cell metastasis, and had enhancing effects on anti-VEGF treatment in MDA-MB-231-established breast cancer xenograft models (153). In 2018, Wei et al. reported the overexpression of AXL in multiple tumor cell lines (including MDA-MB-231, but not MCF-7) and patient-derived samples (154). These researchers also generated AXL-redirected CAR-Ts using a novel AXL-specific scFv, and demonstrated that these CAR-Ts exhibit target antigen-dependent cytotoxicity and cytokine secretion against the AXL-expressing TNBC cell line MDA-MB-231 (154). *In vivo* evaluations of these CAR-Ts in MDA-MB-231-established xenograft models also indicated significant tumoricidal reactions and *in vivo* persistence (154). Moreover, Zhao et al. designed AXL-redirected CAR-Ts co-expressing a constitutively activated IL-7 receptor (C7R), and reported that these CAR-Ts established significant tumoricidal capability, which was higher than that of conventional AXL-redirected CAR-Ts, against TNBC MDA-MB-231 and MDA-MB-468 cell lines (155). These researchers added that the co-expressed C7R significantly enhanced the activation, expansion, and antitumor activity of the developed CAR-Ts (155). Additionally, Zhao et al. indicated that according to *in vivo* results, C7R-co-expressing AXL-redirected CAR-Ts resulted in prolonged survival and a reduced rate of tumor relapse in preclinical mouse models bearing subcutaneous MDA-MB-231 cells (155). Even though such data may underline the applicability of AXL as a CAR-T therapy target antigen in TNBC, further investigations are required.

### Tumor endothelial marker 8 (TEM8)

TEM8, alternatively known as *anthrax toxin receptor 1* (ANTXR1), is an integrin-like transmembrane protein with roles in the migration and invasion of endothelial cells (156). TEM8 is overexpressed in invasive and TNBC breast cancer tissue correlating with elevated possibility and potential of disease relapse in basal breast cancer (157–159). Researchers have demonstrated that overexpressing TEM8 in preclinical breast cancer models resulted in the amplified capability of the tumor for expansion and metastasis whereas blocking or knocking out TEM8 expression hindered tumor progression in various preclinical models (159–162). Byrd et al. generated TEM8-redirected CAR-Ts and assessed the cytotoxicity of these cells *in vitro* and *in vivo* (162). They demonstrated that TEM8-redirected CAR-Ts managed to efficiently inhibit the growth of various TNBC lines (Hs 578T, MDA-MB-231, MDA-MB-436, and MDA-MB-468), a human breast tumor-associated endothelial cell line (HC 6020), and murine tumor-associated endothelial cell lines (2H11 and bEND.3) (162). It is worth mentioning that all of these tumor-associated endothelial cell lines tested positive for variable levels of TEM8 expression (162). Moreover, according to *in vivo* experiments, these researchers indicated that their TEM8-redirected CAR-Ts mediated tumor growth suppression and prolonged survival in localized patient-derived xenograft (PDX) and lung metastatic TNBC cell line LMD231-established xenograft preclinical models and *via* eliminating TEM8-expressing TNBC tumor cells and targeting the tumor endothelium to prevent further tumor neovascularization (162). Moreover, Petrovic et al. generated different types of TEM8-redirected CAR-Ts including one type generated using the same antibody utilized in the discussed study by Byrd et al. (163). The *in vivo* results reported by Petrovic and co-workers indicated that these CAR-Ts rapidly and selectively disappeared from the circulation and caused rapid toxicity after administration into healthy C57BL6 and NSG mice (163). Using TEM8-knockout mouse models, Petrovic et al. demonstrated that the selective clearance of these CAR-Ts from the circulation was because of their cytotoxicity toward normal tissue-expressed TEM8 (163). Such data may speculate critical safety concerns regarding the potential *on-target off-tumor* toxicity in CAR-T-mediated TEM8 targeting in TNBC (163). Therefore, more comprehensive investigations are required before moving on to clinical trials.

### Integrin alpha V beta 3 (α_v_β_3_)

Integrins are adhesion receptors with critical roles in intercellular signal transduction; however, they are also involved in tumor cell migration, tissue invasion, and survival (164). α_v_β_3_ is a well-known integrin in neoplasm-related studies. The expression of this integrin is normally observed in newly forming endothelial cells; however, accumulating evidence confirms its expression in various types of malignancies (165–168). Targeting α_v_β_3_ using antagonists has been evaluated in clinical settings but has not been found effective (169). Moreover, antibody-assisted targeting of this integrin also did not result in beneficial therapeutic effects (170–172). In this regard, researchers have recently contemplated targeting α_v_β_3_ using CAR-Ts.

In 2018, Wallstabe et al. investigated the applicability of targeting α_v_β_3_ using CAR-Ts (173). These researchers used two codon-optimized scFvs as the targeting domain of two different CARs. Of note, these scFvs had been previously humanized (174). The α_v_β_3_-redirected CAR-Ts developed by Wallstabe et al. were also equipped with a truncated epidermal growth factor receptor (EGFRt) enabling the eradication of the infused CAR-Ts by administering the anti-EGFR mAb *cetuximab* (173). In terms of detecting the expression of α_v_β_3_ on human cell lines, these researchers confirmed the high-level expression of this target antigen on the human TNBC cell line MDA-MB-231 as well as on various melanoma cell lines (173). Their data further confirmed the suitability of integrin α_v_β_3_ as a target antigen by being only overexpressed on malignant cells in hematologic and non-hematologic cancers (173). *In vitro* functionality assessments demonstrated that α_v_β_3_-redirected CAR-Ts exhibited potent and exclusive tumoricidal activity by producing IFN-γ and IL-2 upon encountering α_v_β_3_-expressing tumor cells (173). It is worth mentioning that the *in vivo* preclinical assessments of this study did not involve mouse models of TNBC, and the preclinical models in which α_v_β_3_-redirected CAR-Ts demonstrated promising tumoricidal activity were A-375 cell line-established melanoma models (173). Overall, these data only demonstrate that α_v_β_3_ might be a target antigen for CAR-T therapy of TNBC and they cannot guarantee that targeting α_v_β_3_ is safe and effective, and does not result in unwanted toxicities towards normal tissues (173). It is worth noting that CAR-Ts redirected against α_v_β_3_ have also been evaluated for targeting other malignancies such as glioma and it has been indicated that this target antigen holds promising immunotherapeutic value with a low risk of on-target off-tumor toxicity due to its restricted expression on normal tissues (175).

### Receptor tyrosine kinase-like orphan receptor 1 (ROR1)

ROR1 is a type 1 membrane-spanning tyrosine kinase receptor with significant roles in embryonic and fetal development (176, 177). Normally, ROR1 expression is observed during embryogenesis but not in normal adult tissues except for adipocytes and a subset of immature B-cell precursors (176, 177). Malignancy-associated expression of ROR1 has been observed in B-cell chronic lymphocytic leukemia (B-CLL), mantle cell lymphoma (MCL), breast cancer, and ovarian cancer (178).

Various researchers have generated ROR-1-redirected CAR-Ts, and have investigated their various aspects in solid tumors and hematologic malignancies (179–182). In 2019, Wallstabe et al. developed microphysiologic three-dimensional (3D) MDA-MB-231-established breast cancer and A549-established lung cancer models, and reported that ROR1-redirected CAR-Ts efficiently infiltrated into the tumor tissues and mediated tumoricidal reactions against multiple layers of malignant cells (183). These researchers suggested that such 3D models may act as reliable platforms for evaluating the safety and efficacy of CAR-Ts before preclinical and clinical assessments (183). Hudecek et al. are also among other researchers who developed ROR1-redirected CAR-Ts and demonstrated their antitumor activity against various solid tumor cell lines including the breast cancer cell lines MDA-MB-231 and MDA-MB-468 (178). Moreover, Srivastava et al. claimed that ROR1-redirected CAR-Ts mediated lethal bone marrow failure since these cells attack ROR1-expressing stromal cells alongside targeting ROR1-expressing malignant cells (182). In this case, these researchers developed ROR1-redirected CAR-Ts harboring synthetic Notch (synNotch) receptors specific for EpCAM or B7-H3 (expressed by ROR1-expressing tumor cells but not ROR1-expressing stromal cells) and reported that these CAR-Ts safely mediated efficient tumoricidal activity without toxicity (182).

Moreover, a Phase I clinical trial (NCT02706392) has investigated the safety of second generation ROR1-redirected CAR-Ts in patients with various types of neoplasms including metastatic TNBC (184). So far, the reported results of 4 enrolled TNBC patients indicated that dose-limiting toxicities, severe neurotoxicity, or severe cytokine release syndrome (CRS) were not detected at dose levels 1 and 2 (184). It is worth mentioning that half of the patients experienced grade 1 CRS (184). Also, post-CAR-T administration tumor tissue biopsy demonstrated infiltration of CD3-positive T cells and macrophages proposing efficient tumor site CAR-T trafficking (184). In regards to clinical outcomes, 2 out of 4 patients achieved stable disease (one at 15 weeks and the other at 19 weeks post infusion) (184). One patient achieved stable disease after the first CAR-T administration and established partial response following the second CAR-T administration which has prolonged for 14 weeks as of the time of the report (184). Of note, the results of this trial are expected to be updated (184).

### Receptor tyrosine kinase c-Met

c-Met, alternatively known as MET or hepatocyte growth factor receptor (HGFR), is a membrane-bound tyrosine kinase known to have critical roles in organogenesis and cancer development (185). c-Met is involved in the development of TNBC (186). c-Met inhibitors have been studied in various solid tumors and these inhibitors have resulted in encouraging results in lung and ovarian cancer (187, 188). c-Met overexpression has been observed in more than 50% of TNBC patients correlating with unfavorable overall survival (OS) of these patients (185, 186). In this regard, Kim et al. reported that c-Met expression is high in TNBC cell lines, and siRNA-mediated silencing of c-Met decreases the proliferation and migration capacity of certain TNBC cell lines (186). Such data can support the critical role and applicability of c-Met as a target for cancer immunotherapy.

Tchou et al. developed mRNA electroporation-generated second generation c-Met-redirected CAR-Ts and demonstrated the antitumor activity of these cells in *in vitro* killing assays against BT20 (a TNBC-derived breast cancer cell line) and TB129, both with similar c-Met expression levels (189). These CAR-Ts also managed to suppress tumor growth in xenograft preclinical mouse models established using the ovarian cancer cell line SK-OV-3 (189). These researchers also conducted a Phase I clinical trial (NCT01837602) to study the safety and feasibility of intratumoral delivery of these CAR-Ts for treating metastatic breast cancer (189). The results of this trial demonstrated that CAR-T administration was well-tolerated as no CAR-T-related adverse events (>grade 1) were observed (189). Moreover, immunohistochemical analysis of tumors treated with intratumoral CAR-T delivery demonstrated significant administration site tumor necrosis, cellular debris, and the presence of macrophages around the necrotic areas (189). It is worth mentioning that Tchou et al. used mRNA-based CAR-Ts based on the concerns regarding the tumoricidal effects of c-Met-redirected CAR-Ts for targeting non-malignant c-Met-expressing cells (189). Moreover, Shah et al. reported the results from a Phase I clinical trial (NCT03060356) evaluating the safety and feasibility of intravenously delivered mRNA electroporation-generated second generation c-Met-redirected CAR-Ts in patients with metastatic or unresectable melanoma or TNBC with more than 30% c-Met expression (190). According to this report, 5 patients experienced grade 1 or 2 CAR-T infusion-related toxicity (no grade 3 or CRS was observed) but one patient terminated the therapy course due to these toxicities (190). Among the patients with TNBC who received the c-Met-redirected CAR-Ts (4 patients), 2 (50%) achieved stable disease and 2 (50%) experienced partial disease (190). Ultimately, these researchers suggested using lentiviral-generated CAR-Ts alongside lymphodepleting chemotherapy in future clinical studies (190).

### Folate receptor alpha (FRα)

FRα, alternatively termed FOLR1 or folate binding protein (FBP), is a glycosylphosphatidylinositol (GPI)-anchored transmembrane protein with a high affinity for binding the active folate form and managing its transportation (191, 192). The overexpression of FRα has been detected in various solid tumors such as ovarian and breast cancers remarkably correlating with disease grade and stage (193, 194). TNBC is also among solid malignancies in which FRα upregulation has been observed (195). It has been demonstrated that overexpression of FRα provides malignant cells with the privilege of proliferation (195). Song et al. generated FRα-redirected CAR-Ts, and reported target antigen engagement-dependent proinflammatory cytokine secretion by these cells after co-culturing with FRα-expressing human ovarian cell lines in culture (SKOV3, A1847, and OVCAR3) (196). Moreover, these researchers reported that these CAR-Ts induced tumor growth suppression in cell line-established FRα-positive human ovarian cancer preclinical models (196). Years later, Song et al. evaluated FRα-redirected CAR-Ts in breast cancer cell lines and preclinical mouse models (197). These researchers reported that their FRα-redirected CAR-Ts secreted significant levels of IFN-γ upon co-cultivation with TNBC cell lines expressing FRα (197). These researchers also added that their engineered effector cells also mediated remarkable tumor outgrowth suppression in cell line-established preclinical xenograft mouse models of TNBC (197). However, Song et al. concluded that the tumoricidal activity of their FRα-redirected CAR-Ts was not as robust as it was in the case of ovarian cancer xenograft models, where the expression levels of the target antigen were higher (197). Moreover, they demonstrated the same CAR-Ts induced improved tumor suppression in preclinical models established using MDA-MB-231 cells engineered to overexpress FRα (197).

Lanitis et al. used a strategy for generating FRα-redirected CAR-Ts with a reduced level of capability for mediating *on-target off-tumor* toxicity (198). In detail, they generated a *trans-signaling* CAR in which the activation domain is physically separated from the co-stimulatory domain in two separate CARs, one set of which targets mesothelin and the other set targets FRα (198). These CAR-Ts exhibited ineffective cytokine secretion upon encountering target cells expressing only one of the target antigens but exhibited significantly improved cytokine secretion against tumor cells expressing both target antigens *in vitro* (198). Furthermore, these CAR-Ts demonstrated effective tumoricidal activity and persistence *in vivo* (198). Conclusively, these researchers suggested this method as a potent strategy for reducing the possibility of *on-target off-tumor* activity of CAR-Ts towards non-malignant tissues (198). In addition to this strategy, other researchers have also demonstrated that folate-FITC bispecific molecules can manage to mediate the redirection of FITC-redirected CAR-Ts activity against folate receptor (FR)-positive malignant cells (199).

### Epidermal growth factor receptor (EGFR)

EGFR is a membrane-spanning glycoprotein that is a member of the ERBB receptor tyrosine kinase family (200). It has been known to be involved in malignant cell proliferation and metastasis (200). TNBC is among solid tumors in which EGFR overexpression has been detected (201). EGFR targeting using CAR-Ts has been investigated in TNBC as well as other solid tumors (202–204). In detail, Li et al. generated EGFR-redirected CAR-Ts using the non-viral *piggyBac* transposon system, and reported that these cells demonstrated tumoricidal activity against EGFR-positive cells *in vitro* and suppressed tumor growth in human lung cancer xenografts (203). Moreover, a Phase I clinical trial (NCT03182816) has investigated *PiggyBac* transposon-generated EGFR-redirected CAR-Ts in patients with advanced R/R non-small cell lung cancer (202).

In the case of TNBC, Liu et al. investigated EGFR-redirected CAR-Ts *in vitro* and *in vivo* (205). These researchers reported the overexpression of EGFR in TNBC Hs 578T, MDA-MB-468, and MDA-MB-231 cell lines in comparison with the non-TNBC cell line MCF-7 (205). *In vitro* co-cultivation assay of EGFR-redirected CAR-Ts with the mentioned TNBC cell lines demonstrated the target antigen-dependent cytokine secretion and antitumor activity of these CAR-Ts (205). Moreover, these researchers reported tumor growth suppression by EGFR-redirected CAR-Ts in cell line-established and PDX preclinical mouse models (205). Recently, Xia et al. reported the overexpression of EGFR in TNBC cell lines MDA-MB-231, MDA-MB-468, Hs 578T, and HCC1860 (201). These researchers generated third-generation EGFR-redirected CAR-Ts and reported that these effector cells demonstrated specific cytokine secretion, antitumor activity, and upregulation of T-cell activation markers (including CD69 and CD25) upon co-cultivation with EGFR-positive TNBC cells lines (201). *In vivo* assessments using severe combined immunodeficient (SCID) mice subcutaneously implanted with the MDA-MB-231 cell line demonstrated the capability of these CAR-Ts to inhibit TNBC tumorigenesis in preclinical mouse models with “minimal” levels of off‐tumor cytotoxicity (201). Overall, both of the discussed studies proposed that EGFR may be a potent CAR-T target antigen for TNBC treatment; however, more preclinical and clinical investigations are critically required (201).

Tumor-restricted variants of EGFR such as EGFR variant III (EGFRvIII) can be utilized in case of safety concerns regarding the expression of EGFR on normal tissues (206). However, so far, such variants have only been investigated in a limited number of solid tumors such as glioma (206). Moreover, modulating the immunosuppressive nature of the TME using immune checkpoint blockade approaches has been shown to improve CAR-T functioning in solid tumors (207). Several clinical trials (NCT03182816, NCT02873390, NCT02862028, and NCT03170141), some of which are completed and some are still ongoing, have aimed to investigate the effects of EGFR-redirected CAR-Ts with the ability to secrete anti-CTLA-4, anti-PD-1, or anti-PD-L1 antibodies in EGFR-positive advanced solid tumors (207). However, no reports regarding the results of such trials in patients with TNBC have yet been published (207).

### Mesothelin

Mesothelin is a tumor differentiation glycoprotein involved in cell adhesion (208, 209). It has a normally restricted expression on the mesothelial surfaces of the body, but it is remarkably overexpressed in a wide range of solid cancers including TNBC (208, 209). There is scientific evidence that mesothelin is engaged in oncogenesis through various cellular signaling pathways including NF-κB, PI3K, and MAPK (208–210). TNBC is one of the solid tumors in which mesothelin overexpression has been detected. In detail, one study has demonstrated that the majority of TNBC cases (67%) exhibit mesothelin overexpression as confirmed using immunohistochemical analysis (211). The limited normal expression of mesothelin and its high-level expression in a large proportion of TNBC cases have rendered it an appealing target antigen for various types of cancer immunotherapy. In 2019, Del Bano et al. investigated bispecific antibodies with mesothelin targeting and CD16 engagement domains, and demonstrated that this construct mediated the recruitment and penetration of natural killer (NK) cells into tumor spheroids and provoked robust dose-dependent cell-mediated cytotoxicity of mesothelin-expressing TNBC cell lines (212). CAR-T-mediated mesothelin targeting has also been studied in the context of TNBC. In detail, Hu et al. assessed the expression of mesothelin on three TNBC cell lines including MDA-MB-231, BT-549, and Hs 578T (107). They reported that only BT-549 cells (but not MDA-MB-231 and Hs 578T cells) expressed mesothelin as verified by both Western blot and flow cytometry (107). These researchers generated second-generation mesothelin-redirected CAR-Ts and evaluated their performance *in vitro* and *in vivo* (107). Of note, Hu et al. disrupted the PD-1 gene locus in T cells before CAR transgene introduction. In detail, these CAR-Ts demonstrated remarkably increased cytokine production and antitumor activity against PD-L1-expressing cancer cells in culture (107). Accumulating evidence suggests that PD-L1 is remarkably overexpressed in TNBC cells (213). Therefore, the strategy proposed by Hu et al. might overcome the suppressive effects of PD-1-PD-L1 interaction on CAR-Ts (107). Moreover, Hu et al. added that PD-1-deficient mesothelin-redirected CAR-Ts exhibited improved tumor outgrowth suppression and disease recurrence prevention in BT-549-established preclinical TNBC mouse models in comparison with CAR-Ts with or without PD-1-specific antibody blockade (107). Overall, even though more preclinical and clinical data are required for safe conclusions on the applicability of using mesothelin as a CAR-T therapy target antigen for TNBC, the study by Hu et al. highlights the potential of checkpoint blockade alongside mesothelin-redirected CAR-T therapy for targeting TNBC. It is worth mentioning that a Phase I clinical trial (NCT02792114) is currently investigating the safety and tolerability of mesothelin-redirected CAR-Ts in patients with metastatic/advanced mesothelin-proficient breast cancer including TNBC. Moreover, another Phase I/II clinical trial (NCT02414269) is also investigating second generation mesothelin-redirected CAR-Ts in patients with lung cancer or breast cancer. Additionally, two other clinical trials (NCT01355965 and NCT02580747) have been completed but no official reports in regards to the results of these trials in TNBC patients have been published yet.

### Disialoganglioside GD2

GD2 is a surface antigen with normal expression limited to peripheral pain fibers, neurons, and melanocytes (214). GD2 expression has been documented in neuroectoderm-originated neoplasms such as neuroblastoma and melanoma (214). The prevalent tumor cell-restricted expression of GD2 has made it a suitable target antigen for various types of cancer immunotherapy. GD2 is mostly known as a target antigen for the treatment of neuroblastoma. In 2011, Louis et al. conducted a Phase I clinical trial (NCT00085930) by generating GD2-redirected CAR-Ts using Epstein-Barr virus (EBV)-specific cytotoxic T lymphocytes or blood T cells for investigating the effectiveness and prolonged persistence of these cells (215). Back in 2008, these researchers also reported the results of a Phase I clinical trial in 11 pediatric patients with neuroblastoma (216). In 2015, the US FDA approved the anti-GD2 mAb *dinutuximab* (also known as *ch14.18*) for the treatment of pediatric patients with high-risk neuroblastoma (217). This mAb has recently been investigated in TNBC (218, 219). Ly et al. investigated dinutuximab application for targeting GD2-expressing breast cancer stem cells (BCSCs) and suppression of cancer progression (219). BCSCs are a type of cells in the early tumor that are chemotherapy resistant, capable of metastases, and remarkably tumorigenic (218). Therefore, BCSC targeting is known as a critical approach for precluding cancer metastases and rendering tumors susceptible to chemotherapy (219, 220). Ly et al. first demonstrated the upregulation of GD2 in TNBC cell lines, PDX preclinical models, and primary TNBC samples (219). In detail, Hs 578T and HCC1395 were two TNBC cell lines in which more than 90% of cells were GD2-proficient (219). Moreover, GD2 expression was also documented in about 60% of primary TNBC tumors (even though with fluctuating levels) correlating with poorer OS (219). Dinutuximab treatment meaningfully reduced adhesion and migration of MDA-MB-231 and SUM159 TNBC cells and suppressed GD2-upregulated mTOR signaling in BCSCs (219). This mAb also exhibited tumor outgrowth suppression capability in MDA-MB-231-established TNBC xenograft models suggesting the applicability of dinutuximab for TNBC control in the preclinical stage (219).

There are not many studies investigating CAR-Ts targeting GD2 in TNBC. In 2020, Seitz et al. used the scFv derived from ch14.18 to generate GD2-redirected CAR-Ts (221). In detail, TNBC cell lines including MDA-MB-468, MDA-MB-231, Hs 578T, and BT-549 were screened for GD2 expression and it was demonstrated that MDA-MB-231 expressed GD2, even though at very low levels, and Hs 578T and BT-549 exhibited uniform GD2 expression (221). *In vitro* cytotoxicity assay demonstrated these CAR-Ts did not mediate any specific tumoricidal activity towards MDA-MB-231 (221). However, these CAR-Ts induced specific cytotoxicity and cytokine secretion upon co-cultivation with the Hs 578T and BT-549 cell lines (221). Moreover, GD2-redirected CAR-Ts mediated tumor growth suppression in the preclinical orthotopic model of TNBC (established using MDA-MB-231) and inhibited lung metastasis (221). Conclusively, these researchers suggested CAR-T-mediated GD2 targeting as an approach to eradicate disseminated malignant cells and inhibit metastasis (221). However, more preclinical and clinical findings may be required.

### Mucin 1 (MUC1)

MUC1 is a heavily glycosylated membrane-spanning mucin protein expressed on glandular epithelial cells (222–224). The extracellular domain of MUC1 has a variable number tandem repeats (VNTR) region which is rich in serine and threonine residues acting as a platform for the attachment of O-glycans (222–224). O-glycosylation of MUC1 leads to the generation of a tumor-associated aberrantly glycosylated form of MUC1 known as tMUC1 (51, 222–224). tMUC1 is overexpressed in all subtypes of breast cancer, including in 95% of TNBCs (225). Moreover, this antigen has no detectable expression on the cells of normal breast tissues (225). Such characteristics render tMUC1 a great CAR-T therapy target antigen in various types of malignancies including TNBC. To this day, various studies have demonstrated that CAR-T-mediated targeting of the aberrantly glycosylated tumor forms of surface antigens is a safe and feasible approach (51). In this regard, Zhou et al. generated second-generation CAR-Ts using a mAb named TAB004, which is capable of tMUC1 recognition (225). These tMUC1-redirected CAR-Ts demonstrated target antigen-dependent cytotoxicity against nine different TNBC cell lines and released cytokines, chemokines, and granzyme B (225). Moreover, these CAR-Ts inhibited HCC70 tumor outgrowth and maintained their prolonged antitumor activity for HCC70 tumor burden reduction *in vivo* (225). In a nutshell, this study indicated that tMUC1-redirected CAR-Ts harbor significant therapeutic value against tMUC1-positive TNBC with minimal on-target off-tumor toxicity toward normal breast epithelial cells (225).

In addition to t-MUC1, TnMUC1 is another aberrant glycoform of MUC1 that is also highly expressed by TNBC cells (226). Posey et al. generated TnMUC1-redirected CAR-Ts using a Tn-MUC1-specific antibody named 5E5, and reported target antigen-specific antitumor activity of these CAR-Ts against a panel of human primary cells and several cell lines (226). Moreover, these researchers reported that these CAR-Ts managed to suppress tumor growth in T-cell leukemia and pancreatic cancer xenograft *in vivo* models (226). Recently, Zhai et al. reported that their TnMUC1-redirected CAR-Ts generated using Vγ9Vδ2 T cells exhibited *“similar or stronger”* antitumor effects, than CAR-expressing αβ T cells, against breast cancer cell lines with various levels of Tn-MUC1 expression (including T47D, MDA-MB-231, and MDA-MB-468) (227). Similar results were observed upon the co-cultivation of these CAR-Ts with gastric cancer cells (227). However, these researchers indicated that Vγ9Vδ2 TnMUC1-redirected CAR-Ts demonstrated persistence deficiencies for addressing which IL-2 is critical (227). Moreover, Zhai also reported that Vγ9Vδ2 TnMUC1-redirected CAR-Ts had significant tumoricidal activity in gastric cancer preclinical mouse models (227).

It is worth mentioning that clinical trials (NCT04020575 and NCT04025216) are currently evaluating second generation CAR-Ts redirected against cancer glycoforms of MUC1. In an early report from one of these trials (NCT04025216), the researchers indicated that since this study is still in the dose-escalation step and it has completed only 2 of 6 planned dose levels, safety issues or *on-target off-tumor* toxicity of this platform are not yet up for debate (228). Moreover, another clinical trial (NCT02587689) investigating MUC1-redirected CAR-Ts in patients with advanced refractory solid tumors, including TNBC, has been finished. However, no official report of the results of this trial has been published yet.

### Trophoblast cell-surface antigen 2 (TROP2)

TROP2 is a transmembrane protein expressed on the surface of human trophoblast cells (229). The expression of this antigen has been often detected in various epithelium tissue malignancies mediating their tumor-associated behavior and correlating with poor prognosis (229, 230). Such characteristics have rendered TROP2 an attractive target antigen for various types of cancer immunotherapy, especially in TNBC (229). In this regard, a recent study by Liu et al. has reported that TRBAs targeting TROP2 and CD3 suppress tumor growth in both TNBC cell lines and primary tumor cells (231). In detail, the TROP2-CD3 TRBAs developed by these researchers were capable of recruiting T lymphocytes to TROP2-positive tumor cells *in vitro* and into tumor tissues in xenograft TNBC preclinical models (231). The data presented by this study supports the potential of TROP2 for the immunotherapy of TNBC in patients with advanced/metastatic neoplasms (231). Moreover, in 2021, the US FDA approved *sacituzumab govitecan*, a TROP2-redirected mAb conjugated to a topoisomerase I inhibitor drug, for the treatment of patients with R/R unresectable locally advanced or metastatic TNBC who have received two or more prior systemic therapies (232). FDA granted medical use permit to this ADC based on a clinical trial report (NCT01631552) by Bardia et al. which demonstrated that *sacituzumab govitecan* mediated a response rate of 33% (3 complete and 33 partial responses) and a median duration of response of 7.7 months in patients with TNBC who had received a range of 2 to 10 previous lines of therapies (232). In the case of CAR-T-mediated TROP2 targeting, Zhao et al. developed bi-specific TROP2- and PD-L1-redirected CAR-Ts and evaluated their antitumor activity *in vitro* (using TROP2-positive and PD-L1-positive gastric cancer cell lines) and *in vivo* (233). According to the *in vitro* results obtained by these researchers, the tumoricidal activity of bi-specific CAR-Ts was higher than that of mono-specific CAR-Ts (233). These bi-specific CAR-Ts also managed to secrete pro-inflammatory cytokines upon encountering TROP2-positive and PD-L1-positive gastric cancer cells (233). Additionally, *in vivo* results demonstrated that these CAR-Ts remarkably suppressed tumor growth *via* intratumoral injection in preclinical mouse models established using human gastric tumors (233). However, these results only support the suitability of TROP2 as a CAR-T target but not specifically in TNBC CAR-T therapy. In the case of TROP2-redirected CAR-Ts for targeting TNBC, Bedoya et al. generated TROP2-redirected CAR-Ts and reported that these cells demonstrated antitumor activity against TROP2-negative cells in the presence of TROP2-positive cells (234). As claimed by these researchers, this phenomenon has also been observed in the case of CD19-redirected CAR-Ts but it was resolved by the blockade of death receptor ligands; however, Bedoya et al. indicated that this approach only resulted in the partial blockade of TROP2-redirected CAR-T cytotoxicity in this case (234). Moreover, these researchers transferred TROP2-positive tumor exosomes to TROP2-negative tumor cells to elevate the percentage of tumor cells targetable by TROP2-redirected CAR-Ts and to tackle the limitation of antigen heterogeneity in solid tumors (234). In a nutshell, even though CAR-T-mediated TROP2 targeting in TNBC has not been comprehensively investigated, the data discussed here might highlight the potential applicability of this target antigen for the treatment of TNBC.

## The potential toxicities of CAR-T therapy in TNBC

Identifying suitable target antigens is one of the most important steps of CAR-T cell therapy in solid tumors. “On-target off-tumor” effect is a CAR-T cell-mediated adverse event that takes place when a CAR-T-redirected target antigen is present (even at very low levels) on normal tissues, especially vital organs. The best strategy to prevent this adverse event is to select target antigens that are completely absent from normal tissues or have undetectable expression levels. However, this strategy is quite unpractical since every target antigen, which is overexpressed on malignant tissue cells, can also be detected in normal tissues. Therefore, researchers have focused on strategies that can be beneficial for preventing or reducing the occurrence of such events. For instance, “safety switches” which include suicide genes have been utilized in the construct of CAR-Ts for targeting TNBC (173). In detail, Wallstabe et al. have studied the applicability of α_v_β_3_-redirected CAR-Ts equipped with EGFRt which allows the eradication of the infused CAR-Ts using the anti-EGFR mAb *cetuximab* (173). Furthermore, other novel CAR designs such as dual CARs and synNotch CARs have also been investigated in solid tumor CAR-T therapy for preventing off-tumor toxicities (182). Researchers have claimed that ROR1-expressing stromal cells are simultaneously targeted by ROR1-redirected CAR-Ts in the context of targeting ROR1-expressing malignant cells (182). This “on-target off-tumor” toxicity can lead to lethal bone marrow failure; therefore, an efficient strategy is required for preventing this adverse event. Srivastava et al. generated ROR1-redirected CAR-Ts with synNotch receptors specific for EpCAM or B7-H3 (182). Conclusively, these researchers added that such CAR-Ts mediated efficient antitumor responses without causing toxicity (182). In addition to these strategies, *trans-signaling* CARs are also considered innovative in terms of preventing on-target off-tumor toxicities (198). In the context of TNBC, Lanitis et al. generated trans-signaling FRα-redirected CAR-Ts in which the CAR activation domain was physically detached from the CAR co-stimulatory domain and they were incorporated into two separate CARs one targeting mesothelin and the other targeting FRα (198). These CAR-Ts were evaluated in preclinical assessments, and it was proposed that this strategy can decrease the risk of on-target off-tumor toxicity against normal tissues (198).

In the context of CAR-T therapy of hematologic malignancies, such as those targeting CD19, due to the expression of CD19 by both normal cells and malignant blasts, CAR-T therapy results in the elimination of both of these cells leading to a phenomenon known as “*B-cell aplasia*” (235). This occurrence is a marker of an efficient CAR-T therapy of hematologic malignancies, even though it makes the relative patients susceptible to opportunistic infections to tackle which the patients should undergo immunoglobulin replacement (235).

As mentioned earlier, the application and efficiency of CAR-Ts whose scFv targeting domains have been subjected to affinity-tuning have also been studied (52). Such CAR-Ts can be favorable in distinguishing between tumor cells overexpressing the target antigen and normal cells expressing the antigen at physiologic levels (52). In this regard, Park et al. demonstrated that affinity-tuned ICAM-1-redirected CAR-Ts are capable of targeting malignant cells with ICAM-1 overexpression while sparing normal cells expressing ICAM-1 at basal physiologic levels (52). Moreover, mRNA-based CAR-Ts have also been proposed as a platform for preventing on-target off-tumor toxicities. In this regard, Tchou et al. developed mRNA-based c-Met-redirected CAR-Ts to avoid CAR-T-mediated targeting of c-Met-expressing normal cells (189). They reported that these cells demonstrated efficient antitumor activity against TNBC both *in vitro* and *in vivo* (189). These researchers also evaluated the safety and efficacy of these CAR-Ts in a Phase I clinical trial (NCT01837602) (189).

CRS is well-known CAR-T cell therapy adverse event which is more likely to occur in CAR-T therapy of hematologic malignancies (235, 236). CRS results from the rapid activation of various immunological pathways. Its critical damages can include cardiac-related toxicities and hyponatremia. CRS has also been detected in patients with solid tumors undergoing CAR-T therapy (236). As discussed throughout the article, there are not many clinical trials investigating CAR-T therapy for the treatment of patients with TNBC. Some of these trials have been completed and some are still ongoing. Among the trials that have been completed, only a few of them have reported their findings. In detail, in a Phase I clinical trial (NCT02706392) studying ROR1-redirected CAR-Ts in patients with various solid tumors including metastatic TNBC, it was reported that out of 4 TNBC patients, half of them experienced grade 1 CRS (184). According to a report by Tchou et al. from a Phase I clinical trial (NCT01837602) assessing the safety and feasibility of intratumoral delivery of c-Met-redirected CAR-Ts in patients with metastatic breast cancer, it was reported that the CAR-Ts were well-tolerated and no CAR-T-associated toxicities (> grade 1) were documented (189). Moreover, in another a Phase I clinical trial (NCT03060356) investigating the same target antigen, it was reported that 5 patients experienced grade 1 or 2 CAR-T delivery-associated adverse events (no grade 3 or CRS were reported) (190).

CRS is a medical condition that demands rapid clinical intervention to prevent the condition from worsening. Generally, antihistamines or corticosteroids are recommended for low-grade CRS management (236). However, in the case of CAR-T therapy-mediated CRS, more efficient strategies are required, especially in the case of hematologic malignancies such as B-cell acute lymphoblastic leukemia (B-ALL) (235). IL-1 and IL-6 blockade, GM-CSF blockade, antibody-based immunotherapy pretreatment, therapeutic plasma exchange, hemofiltration, and fractionated CAR-T infusion are among strategies proven to be efficient in the management of severe lethal CRS after CAR-T therapy (235, 237–245). Such strategies can also be applied in the case of solid tumor CAR-T therapy including TNBC CAR-T therapy.

## Conclusion

CAR-T products have been available on the market as a treatment option for R/R hematologic malignancies in the past recent years. However, CAR-T-mediated targeting of solid tumors or some hematologic malignancies, such as T-cell neoplasms, face additional unexpected challenges that remarkably restrain the antitumor potential of these therapeutics. TNBC is known as a heterogeneous breast cancer subtype that is mainly resistant to conventional therapies. The immunogenic nature of this neoplasm has proven that immunotherapy-based therapeutics can lead to favorable clinical benefits. For instance, the promising clinical outcomes of the checkpoint inhibitor *atezolizumab* in combination with *nab-paclitaxel* led to its approval by the US FDA for the treatment of locally or metastatic advanced unresectable TNBC (18). However, CAR-T therapy of TNBC is in an emerging field as it is focused on discovering the suitable and targetable TAAs, mostly in preclinical and early clinical stages. Of note, to overcome the hurdles of CAR-T therapy in TNBC, various challenges of solid tumor CAR-T therapy should be tackled beforehand. Various critical stratagems should be employed to ensure the safety and effectiveness of CAR-T therapy in solid tumors. Combinatorial CAR-T therapy with other types of therapies can result in improved clinical outcomes of CAR-T therapy in solid tumors (70). For instance, ECM- or CAF-targeting agents can be applied for maximizing CAR-T antitumor effects (70). Also, macrophage- or monocyte-eliminating agents are beneficial for amplifying the tumoricidal impact of CAR-Ts, or anti-angiogenic agents can be leveraged for enhancing the tumor site trafficking of CAR-Ts (70). Moreover, other types of effector cells have also been investigated for the expression of CARs. In this regard, γδ-CAR-Ts and CAR-expressing NK cells (CAR-NKs) have been investigated for the treatment of both hematologic and solid malignancies, including TNBC (68, 246–248). Such alternative CAR-expressing effector cells might be beneficial for tackling various types of CAR-T therapy challenges (68, 246–248).

Throughout this article, we reviewed various novel CAR-T target antigens for the selective targeting of TNBC. A number of these target antigens have only been evaluated in *in vitro* and *in vivo* studies while some of them have found their way to in-human clinical investigations, as summarized in **Table 3**. Moreover, in addition to the discussed target antigens, new targets can also be discovered. For instance, stage-specific embryonic antigen-4 (SSEA-4) is among more novel antigens detected in a proportion of TNBCs, and CAR-Ts against this target antigen have been investigated *in vitro* and *in vivo* (249). However, such antigens are not comprehensively studied as TNBC CAR-T-targetable antigens and require more in-depth assessments.

**Table 3:** A summary of different clinical trials investigating CAR-Ts against different target antigens for the treatment of TNBC and other solid tumors.

Target antigen	ClinicalTrials.gov identifier	Phase	Participants	Source	Start-completion date
NKG2D ligand	NCT04107142	I	10	Allogeneic	2019-2021
ROR1	NCT02706392	I	21	Autologous	2016-2021
c-Met	NCT01837602	I	6	Autologous	2013-2018
	NCT03060356	Early I	77	Autologous	2016-2020
Mesothelin	NCT01355965	I	18	Autologous	2011-2015
	NCT02580747	I	20	Autologous	2015-2018
	NCT02792114	I	186	Autologous	2016-2023
	NCT02414269	I/II	113	Autologous	2015-2024
MUC1	NCT02587689	I/II	20	Autologous	2015-2018
A cleaved form of MUC1	NCT04020575	I	69	Autologous	2020-2035
TnMUC1	NCT04025216	I	112	Autologous	2019-2036

In a nutshell, it is safe to mention that considerable efforts have been made in the field of TNBC CAR-T therapy as demonstrated by several ongoing preclinical and clinical studies. However, there is still a long way to go for some of the proposed strategies (for enhancing the efficacy of CAR-T therapy in solid tumors) and several TNBC target antigens discussed in this article. Successful CAR-T therapy in TNBC is substantially reliant on improving the specificity, safety, and efficacy of CAR-T therapy in solid tumors by both choosing the most appropriate target antigens and addressing the unmet restricting challenges. Until then, fingers are crossed for CAR-T therapy to be able to act as an efficient treatment approach alongside the conventional TNBC treatment modalities.

## Author contributions

FN: Investigation, writing - original draft, validation. MK: Writing - original draft. SMJM: Writing - original draft. MMK: Writing - original draft. MAN: Conceptualization, writing - review & editing. FS: Conceptualization, writing - review & editing. SDS: Writing - original draft. SEB: Writing - original draft. PouSK: Conceptualization, investigation, writing - original draft, writing - review & editing, validation, supervision. PooSK: Conceptualization, investigation, writing - original draft, writing - review & editing, validation, supervision. All authors contributed to the article and approved the submitted version.

## Funding

This research did not receive any specific grant from funding agencies in the public, commercial, or not-for-profit sectors.

## Conflict of interest

The authors declare that the research was conducted in the absence of any commercial or financial relationships that could be construed as a potential conflict of interest.

## Publisher’s note

All claims expressed in this article are solely those of the authors and do not necessarily represent those of their affiliated organizations, or those of the publisher, the editors and the reviewers. Any product that may be evaluated in this article, or claim that may be made by its manufacturer, is not guaranteed or endorsed by the publisher.
